# SnRK1 Kinase and the NAC Transcription Factor SOG1 Are Components of a Novel Signaling Pathway Mediating the Low Energy Response Triggered by ATP Depletion

**DOI:** 10.3389/fpls.2019.00503

**Published:** 2019-05-10

**Authors:** Hidefumi Hamasaki, Yukio Kurihara, Takashi Kuromori, Hiroaki Kusano, Noriko Nagata, Yoshiharu Y. Yamamoto, Hiroaki Shimada, Minami Matsui

**Affiliations:** ^1^Synthetic Genomics Research Group, RIKEN Center for Sustainable Resource Science, Yokohama, Japan; ^2^Department of Biological Science and Technology, Tokyo University of Science, Tokyo, Japan; ^3^Applied Biological Sciences, Gifu University, Gifu, Japan; ^4^Gene Discovery Research Group, RIKEN Center for Sustainable Resource Science, Yokohama, Japan; ^5^Department of Chemical and Biological Sciences, Faculty of Science, Japan Woman’s University, Tokyo, Japan

**Keywords:** ATP, hypocotyl elongation, mitochondria, SOG1, SnRK1 kinase

## Abstract

Plant growth is strictly controlled by cell division, elongation, and differentiation for which adequate supplies of intracellular ATP are required. However, it is unclear how changes in the amount of intracellular ATP affect cell division and growth. To reveal the specific pathway dependent on ATP concentration, we performed analyses on the *Arabidopsis* mitochondria mutation *sd3*. The mutant is tiny, a result of a low amount of ATP caused by the disruption of Tim21, a subunit of the TIM23 protein complex localized in the inner membrane of the mitochondria. Loss of function of *suppressor of gamma response 1* (*SOG1*) also restored the dwarf phenotype of wild type treated with antimycin A, a blocker of ATP synthesis in mitochondria. The *sd3* phenotype is partially restored by the introduction of *sog1, suppressor of gamma response 1*, and *kin10*/*kin11*, subunits of Snf1-related kinase 1 (SnRK1). Additionally, SOG1 interacted with SnRK1, and was modified by phosphorylation *in planta* only after treatment with antimycin A. Transcripts of several negative regulators of the endocycle were up-regulated in the *sd3* mutant, and this high expression was not observed in *sd3sog1* and *sd3kin11*. We suggest that there is a novel regulatory mechanism for the control of plant cell cycle involving SnRK1 and SOG1, which is induced by low amounts of intracellular ATP, and controls plant development.

## Introduction

In multi-cellular organisms, DNA replication and cell division, expansion and differentiation are strictly controlled by cell cycle progression. In these processes ATP acts as a major energy substrate (Bártová et al., 2004; Xiong et al., 2012). Decreasing the amount of intracellular ATP affects cell survival. ATP is produced mainly in the mitochondria (Robison et al., 2009). It is reported that it controls not only cell cycle progression, but also regulation of metabolism, biosynthetic reactions and enzyme activities throughout the plant (Hardie, 2004). A dysfunction in mitochondrial activity causes a decrease in intracellular ATP and results in growth arrest of the cell. We previously reported that mutations in mitochondrial proteins cause inhibition of hypocotyl elongation in darkness (Kumar et al., 2012; Hamasaki et al., 2012). In Arabidopsis, hypocotyl elongation is mainly controlled by the endocycle, especially in darkness, and CyclinA2;1 and A2;3 are key regulators of the endocycle (Imai et al., 2006; Yoshizumi et al., 2006). The cell number making up the cell file in the hypocotyl is the same in both light- and dark-grown seedlings, and the length of each cell is correlated with the ploidy level of the cell. The *tim50* mutant shows a short hypocotyl when grown in darkness. *AtTIM50* encodes a member of the TIM23 protein complex localized in the inner membrane of mitochondria (Kumar et al., 2012). The mitochondrion is composed of two layers of membranes. TIM23 protein complex is localized in the inner membrane and is involved in the transport of nuclear-encoded mitochondrial proteins. *sd3* is another mutant with short hypocotyls when grown in the dark, and is seedling lethal in white light (Hamasaki et al., 2012). *SD3* encodes a Tim21 homolog that is another possible subunit of the TIM23 protein complex. Although mitochondrial activity is important for proper development, especially for hypocotyl elongation, its signal transduction to control plant growth is still unclear.

Snf-related kinase 1 (SnRK1) is a plant homolog of mammalian AMP-activated protein kinase (AMPK) and the main component of stress and energy signal transduction in plants (Baena-González and Sheen, 2008; Crozet et al., 2014). AMPK is a protein complex comprising three subunits and functions in the regulation of energy and carbon metabolism. In mammals, AMPK is known to be activated by the AMP/ATP ratio (Wilson et al., 1996; Hardie et al., 1998; Polge and Thomas, 2007). It is known that AMP interact to between α and γ subunit of AMPK. Therefore, AMPK function as energy sensor. However in plant, it is assumed that SnRK1 is not directly regulated by AMP because there is not AMP interaction site in any SnRK1 complex. Since, previous studies have shown that SnRK1 dephosphorylation is inhibited by AMP (Sugden et al., 1999). Also, SnRK1 is activated in starvation of inorganic phosphate (Pi). Inorganic phosphate affects reduction in ATP (Fragoso et al., 2009). Thus, SnRK1 may function as an indirectly energy sensor.

SnRK family is composed of SnRK1 and two other subfamilies, SnRK2 and SnRK3 in plants. Those are shown less sequence similarity with SNF1, unlike SnRK1. SnRK2 and SnRK3 are unique to plants and are mostly involved in abscisic acid (ABA) and environmental stress signaling (Dong et al., 2012; Crozet et al., 2014). SnRK1 controls global metabolic regulation in response to nutritional and environmental stresses such as flooding (Hardie, 2004; Halford and Hey, 2009; Coello et al., 2011; Cho et al., 2012).

KIN10 and KIN11 are the catalytic subunits of SnRK1 and essential to SnRK1-mediated signal transduction. Previous work has revealed that plants over-expressing *KIN10* or *KIN11* showed reduction in shoot and root growth, and delayed flowering (Baena-González et al., 2007; Tsai and Gazzarrini, 2012; Simon et al., 2018). Hence, the catalytic subunit of SnRK1 is responsible for signal transduction for plant growth and development.

*Suppressor of gamma response 1* (*SOG1*) encodes a NAC domain transcription factor and the *sog1* mutant was first isolated as a revertant of the mutant defective in the DNA repair endonuclease XPF. SOG1 transmits the signal for developmental arrest caused by DNA damage through exposure to high γ-irradiation (Preuss and Britt, 2003; Yoshiyama et al., 2009). Also, SOG1 is suggested to function in signal transduction for the control of the cell cycle by external stimuli of the endocycle (Adachi et al., 2011; Yi et al., 2014). Phosphorylation of SOG1 is crucial for the transient arrest of the cell cycle (Yoshiyama et al., 2013).

These observations described above suggest that SnRK1 and SOG1 play a role in the response to a low amount of ATP in plants. Here, we investigate whether there is a low ATP signal pathway in plants and which components are involved in this signal transduction. We find that the amount of intracellular ATP can act as a signal as well as an energy source, and that the intracellular ATP signal is transduced by SnRK1 and SOG1 to control of several cell cycle-related genes.

## Results

### *kin10* and *kin11* Mutations Partially Rescue Seedling-Lethal Phenotype of *sd3* Under White Light

**FIGURE 1:**
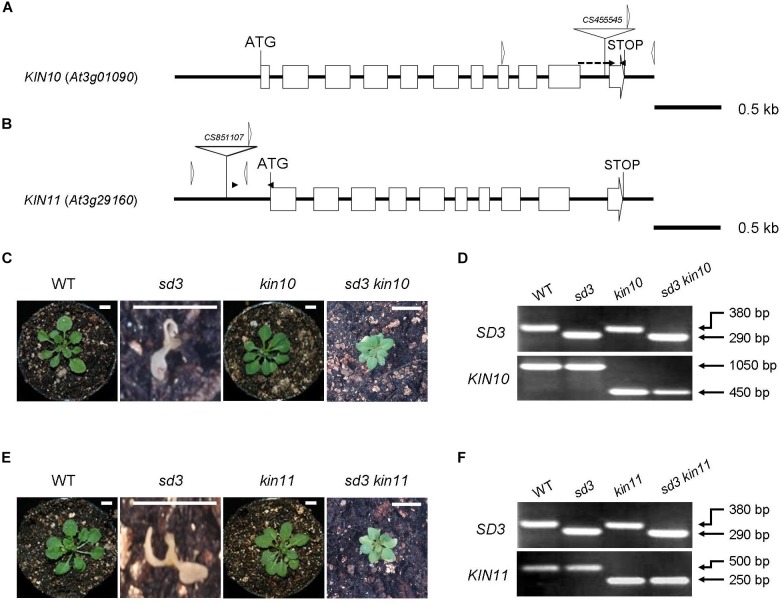
Severe dwarf morphology of *sd3* grown in light was partially rescued by introduction of *kin10* and *kin11* mutations. **(A)** Schematic illustration of the *KIN10* gene. The white boxes and solid lines indicate exons and introns, respectively. The white triangle indicates the *T*-*DNA* insertion site of *kin10.* The black arrowheads indicate the positions of the primers used for real-time RT-PCR in Supplementary Figure S1A. The forward primer was designed to ‘sandwich’ the intron. The white arrowheads indicate the positions of the primers used for the genotyping in Figure 2D. The genotyping primers and real-time RT-PCR primers are specified in Supplementary Table S1. **(B)** Schematic illustration of the *KIN11* gene. The white boxes and solid lines indicate exons and introns, respectively. The white triangle indicates the *T*-*DNA* insertion site of *kin11.* The black arrowheads indicate the positions of the primers used for real-time RT-PCR in Supplementary Figure S1B. The white arrowheads indicate the positions of the primers used for the genotyping in Figure 1F. **(C)** Photographs show the phenotypes of 35-day-old light-grown seedlings. From left, WT, *sd3, kin10*, and *sd3kin10* are shown. **(D)** Upper and bottom panels show PCR bands for genotyping *SD3* (upper) and *KIN10* (bottom), respectively. **(E)** Photographs show phenotypes of 35-day-old light-grown seedlings. From left, WT, *sd3, kin11*, and *sd3kin11* are shown. **(F)** Upper and bottom panels show PCR bands for genotyping *SD3* (upper) and *KIN11* (bottom), respectively. **(C,E)** Scale bar: 0.5 cm. The genotyping primers and real-time RT-PCR primes are specified in “Materials and Methods” section and in Supplementary Table S1, respectively. **(D,F)** The PCR products of *SD3, KIN10* and *KIN11* were separated on a 1.2% agarose gel.

Our previous paper has shown that addition of compound C, which is a potent inhibitor of AMPK, partially rescues the short hypocotyl phenotype of the *tim50* mutant in darkness (Kumar et al., 2012). We speculated that disruption of SnRK1 function partially rescues the short hypocotyl phenotype of the *sd3* mutant. To investigate this, we examined the *KIN10* and *KIN11* mutants. The *kin10*-*1* and *kin11*-*1* mutants have T-DNA insertions in the 10th intron and the promoter region, respectively (Figure 1A,B). Quantitative reverse transcription (RT)-PCR analysis revealed that accumulation of transcripts was very low in *kin10-1* and almost undetectable in *kin11-1* mutants (Supplementary Figures S1A,B). A previous report showed that loss of function of either *KIN10* or *KIN11* did not result in an abnormal phenotype, probably due to functional redundancy (Baena-González et al., 2007). As expected, *kin10-1* and *kin11-1* mutants grown in white light did not show any obvious differences compared to WT during a 35-day growth period (Figure 1C,E). We were unable to generate a *kin10kin11* double mutant, suggesting that it is embryonic lethal. *kin10-1* and *kin11-1* mutants were crossed with the *sd3* mutant to generate *sd3kin10* and *sd3kin11* double mutants (Figure 1C–F). When these mutants were grown in white light neither showed any growth arrest at the seedling stage and they developed true leaves, while *sd3* showed arrested growth at the seedling stage (Figure 1C,E). Both *sd3kin10* and *sd3kin11* grew to maturity and *sd3kin11* later produced seeds (Supplementary Figures S3A,B,D). The growth rate of both *sd3kin10* and *sd3kin11* was about half that of wild type (Supplementary Figures S3E,F) and *sd3kin11* required 4 months to set seed.

### Overexpression of *KIN10* and *KIN11* Causes Short Hypocotyls in Dark

**FIGURE 2:**
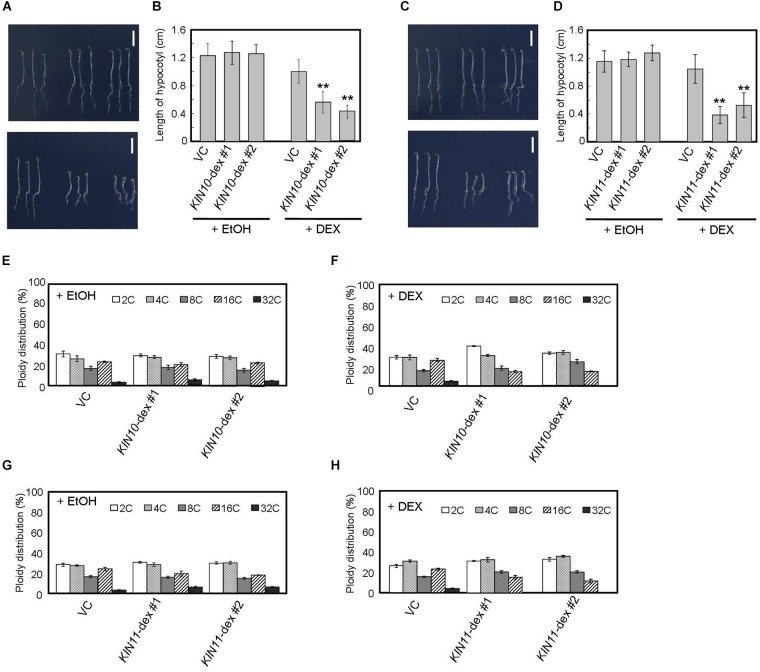
DEX-inducible *KIN10* and *KIN11* over-expression plants have decreased polyploidy levels in dark. **(A,B)** Phenotype of 5-day-old seedlings of vector control (VC), *KIN10*-dex #1, and *KIN10*-dex #2 grown on medium containing EtOH (**A**, upper panel) and 2 mg/ml DEX (**A**, bottom panel) in the dark. **(B)** The average of hypocotyl length of vector control (VC), *KIN10*-dex #1, and *KIN10*-dex #2. 80–100 seedlings were measured. **(C,D)** Phenotypes of 5-day-old seedlings of vector control (VC), *KIN11*-dex #1, and *KIN11*-dex #2 grown on medium containing EtOH (**C**, upper panel) and 2 mg/ml DEX (**C**, bottom panel) in the dark. **(D)** The average of hypocotyl length of the vector control (VC), *KIN11*-dex #1, and *KIN11*-dex #2. 80–100 seedlings were measured. **(B,D)** Error bars indicate standard deviation. Asterisks indicate significance at *P* < 0.05 by *t*-test when compared to VC. **(E,F)** Relative ratio of each ploidy of dark-grown hypocotyls of VC, *KIN10*-dex #1, and *KIN10*-dex #2 grown on medium containing EtOH **(E)** and 2 mg/ml DEX **(F)** in the dark. Approximately 20 seedlings were used in ploidy analysis. **(G,H)** Relative ratio of each ploidy of dark-grown hypocotyls of VC, *KIN11*-dex #1, and *KIN11*-dex #2 grown on medium containing EtOH **(G)** and 2 mg/ml DEX **(H)** in the dark. Approximately 20 seedlings were used in ploidy analysis. **(E–H)** Error bars indicate standard error of five biological replicates.

From the observation that *kin10* and *kin11* substantially rescue the seedling-lethal phenotype of *sd3* (Figure 1), we speculated that *KIN10* and *KIN11* play a role in the signal from the mitochondria that causes suppression of plant growth. We generated 16 and 10 transgenic plants that overexpress *KIN10* (*KIN10*-dex) and *KIN11* (*KIN11*-dex), respectively, upon induction with dexamethasone. We selected two independent lines for each transgene. As expected, expression of both *KIN10* and *KIN11* was increased by DEX in these transgenic lines (Supplementary Figures S2A,B). It is reported that light-grown seedlings overexpressing *KIN10* showed delayed growth and onset of senescence (Baena-González et al., 2007). Also, plants overexpressing HA-tagged *KIN10* showed delayed germination, flowering and senescence (Tsai and Gazzarrini, 2012). As expected, dark-grown 5-day-old *KIN10*-dex or *KIN11*-dex plants, when continuously treated with DEX, showed short hypocotyls in darkness (Figure 2A–D).

*Arabidopsis* hypocotyls are composed of cells of several ploidies and hypocotyl elongation is caused by growth of individual cells with high ploidy (Gendreau et al., 1997). Therefore, such hypocotyl cells are important indicators for growth in the dark. We performed flow cytometric analysis on hypocotyl cells of 5-day-old dark-grown seedlings and showed that, after DEX treatment, the fractions of 16C and 32C cells decreased in both *KIN10* and *KIN11* overexpressing plants (Figure 2E–H). These results suggest that *KIN10* and *KIN11* negatively control plant growth by decreasing ploidy level.

### *sog1* Shows Resistance to Antimycin A

**FIGURE 3:**
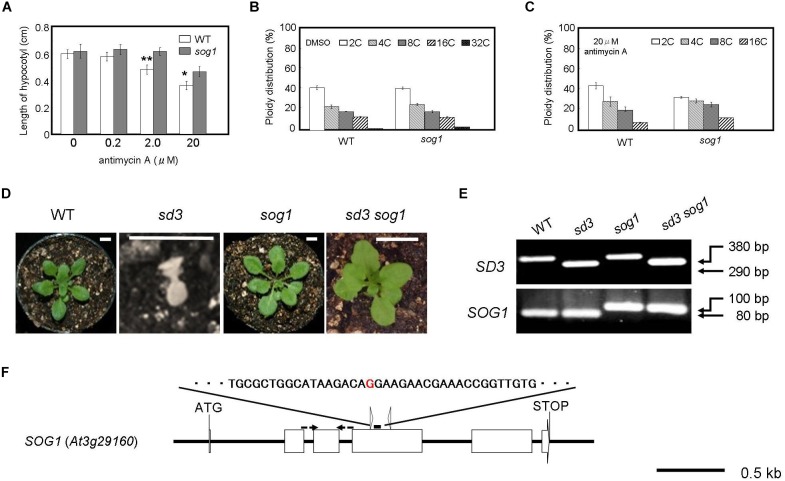
Severe dwarf morphology of *sd3* in light was partially rescued by introduction of *sog1*. **(A)** Schematic illustration of the *SOG1* gene and point mutation site. The red character indicates the point mutation (G to A). The point mutation mutant produces a 100 bp PCR fragment and WT an 80 bp fragment after digestion with ScrFI (New England BioLabs). **(B)** Phenotype of 3-day-old seedlings of WT (Col-0) and *sog1* grown on medium containing 0 (DMSO), 0.2, 2.0, and 20 μg/ml antimycin A in the dark. Approximately 80 seedlings were measured. Error bars indicate standard deviation. **(C,D)** Relative ratio of each ploidy of cells of 3-day-old seedlings of WT (Col-0) and *sog1* grown on medium containing 0 (DMSO) **(C)** and 20 μg/ml antimycin A **(D)** in the dark. Approximately 20 seedlings were used for ploidy analysis. Error bars indicate standard deviation of five biological replicates. Asterisks indicate significance at *P* < 0.05 by *t*-test when compared to *sog1*. **(E)** Photographs show phenotypes of 35-day-old light-grown seedlings. From left, WT, *sd3, sog1*, and *sd3sog1* are shown. Scale bar: 0.5 cm. **(F)** Upper and bottom panels show PCR bands for genotyping *SD3* and *sog1*, respectively. The genotyping primers are specified in Supplementary Table S1. The PCR products of *SD3* and *SOG1* were separated on 1.2% and 3% agarose gels, respectively.

To identify components of the signal transduction from mitochondria, we focused on the *SOG1* gene because SOG1 is suggested to function in the signal transduction for the control of the cell cycle and endocycle (Adachi et al., 2011; Yoshiyama et al., 2017). The *sog1* mutant has a G to A substitution in the genomic sequence resulting in a G155R amino acid change in the NAC domain (Figure 3A) (Yoshiyama et al., 2009). We tested whether this mutant’s growth is affected by mitochondrial dysfunction.

When the mutant is grown in the dark for 3 days in the presence of antimycin A, it is more resistant to the chemical in terms of the reduction in hypocotyl length compared to WT (Figure 3B). Antimycin A is a chemical that causes dysfunction of mitochondria similar to the *sd3* mutation. At a concentration of 20 μM antimycin A, the reduction in polyploidy was weaker in *sog1* compared to WT (Figure 3C,D). These results imply that SOG1 is involved in the signaling resulting from a low amount of ATP from the mitochondria.

### *sog1* Mutation Also Partially Rescues Seedling-Lethal Phenotype of *sd3* Under White Light

The *SOG1* mutation partly suppresses the hypocotyl elongation inhibition caused by antimycin A (Figure 3B). To check whether loss of function of *SOG1* can suppress the mutant phenotype of *sd3*, we generated the *sd3sog1* double mutant. The *sog1* mutant does not show any abnormal phenotype when grown under white light for 35 days (Supplementary Figures S3C,D). *sd3sog1* double mutant also did not show the same growth arrest as the *sd3* single mutant (Figure 3E,F). *sd3sog1* produces true leaves, continues to develop and sets seeds (Supplementary Figures S3C–F). The growth rate of *sd3sog1* is much slower than that of WT (Supplementary Figures S3E,F).

### *KIN11* and *SOG1* Suppresses *sd3’s* Shorter Hypocotyls in Dark

**FIGURE 4:**
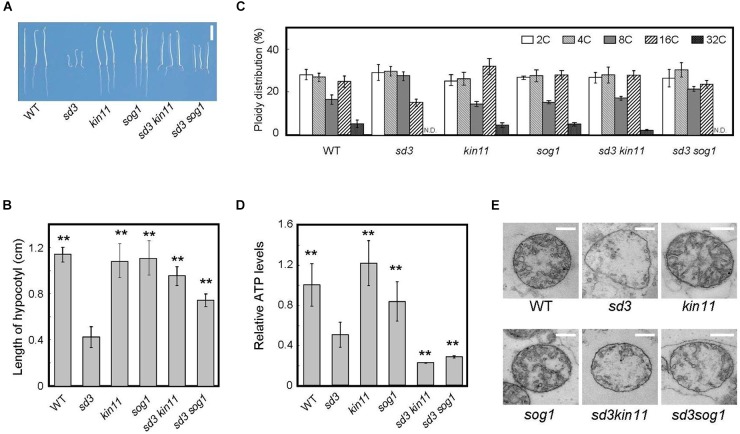
Phenotype of 5-day-old dark-grown *sd3* seedlings showing short hypocotyls with decrease in polyploidy level was partially rescued by introduction of *kin11* and *sog1.* **(A)** Phenotypes of 5-day-old dark-grown seedlings of WT, *sd3, kin11, sog1, sd3kin11*, and *sd3sog1*. Scale bar: 0.5 cm. **(B)** Hypocotyl lengths of 5-day-old dark-grown seedlings of WT, *sd3, kin11, sog1, sd3kin11*, and *sd3sog1.* 40–50 seedlings were measured. Error bars indicate standard deviation. **(C)** Relative ratio of each ploidy of 5-day-old dark-grown hypocotyls of WT, *sd3, kin11, sog1, sd3kin11*, and *sd3sog1*. Approximately 20 seedlings were used for ploidy analysis. Error bars indicate standard error of three biological replicates. **(D)** Total amount of ATP accumulation in 5-day-old dark-grown seedlings of WT, *sd3, kin11, sog1, sd3kin11*, and *sd3sog1.* Approximately 20 seedlings were used for ATP measurements. Error bars indicate standard error of three biological replicates. **(B,D)** Asterisks indicate significant differences compared with *sd3* (Student’s *t*-test, *P* < 0.05). **(E)** Mitochondria in the intact hypocotyls of 5-day-old dark-grown WT, *sd3, kin11, sog1, sd3kin11, sd3sog1*. Scale bar: 400 nm.

We examined whether inhibition of hypocotyl elongation in *sd3* is suppressed by *kin11* or *sog1* in darkness. We were unable to look at *kin10* because *sd3kin10* does not set seeds. Five-day-old seedlings of both *sd3kin11-1* and *sd3sog1* mutants grown in the dark showed longer hypocotyls compared to *sd3* (Figure 4A,B). In particular, the hypocotyl length of the *sd3kin11-1* double mutant was almost the same as that of WT. Ploidy fractions of hypocotyl cells in *sd3kin11-1* were almost the same as WT (Figure 4C), whereas the *sd3sog1* mutants showed an increase in the 16C cell fraction compared to *sd3* (Figure 4C).

The amount of intracellular ATP was measured using a firefly bioluminescence assay (Hamasaki et al., 2012). When grown in the dark, the total amount of intracellular ATP was lower in *sd3* and much lower in the *sd3kin11-1* and *sd3sog1* double mutants compared to WT (Figure 4D). This may be because the signal to arrest cell growth, induced by the low amount of intracellular ATP, is blocked by the *kin11-1* or *sog1* mutation. Therefore, the plant’s growth is forced to progress, resulting in dark-grown seedlings of these double mutants expending most of their ATP. This observation suggests that the amount of ATP is a signal for plant growth, and is not just for energy.

The mitochondria in *sd3* were greatly deformed, having poor cristae compared with WT. Those in *sd3kin11* and *sd3sog1* were also deformed (Figure 4E), implying that the distorted function of the mitochondria in *sd3* is maintained in the *sd3kin11* and *sd3sog1* mutants.

### KIN10 and KIN11 Interact With SOG1

**FIGURE 5:**
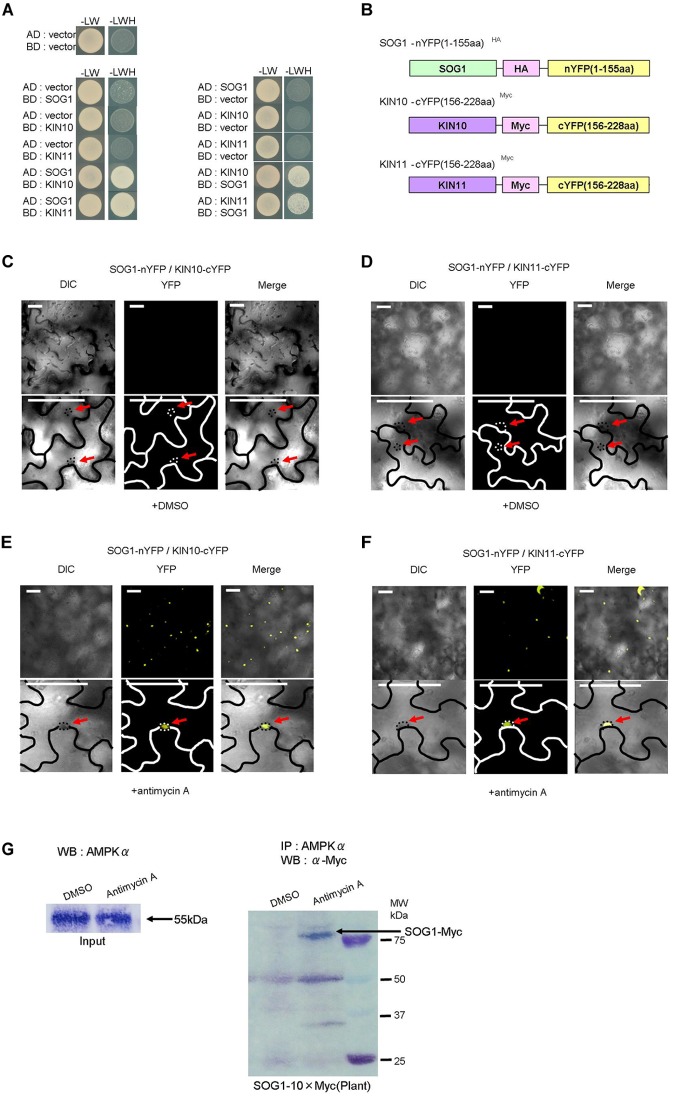
SOG1 directly interacts with KIN10/11 in Y2H and *in planta*. **(A)** Yeast two-hybrid (Y2H) assays show the interaction between SOG1 and KIN10, and SOG1 and KIN11. The vectors containing only AD (the activate domain) or BD (the LexA DNA-binding domain) act as negative controls. SOG1, KIN10, and KIN11 bait constructs were fused to the LexA DNA-binding domain and SOG1, KIN10, and KIN11 prey constructs were fused to the activation domain. **(B)** Schematic diagrams of SOG1 and KIN10/11 domains and constructs used in bi-molecular fluorescence complementation (BiFC). **(C–F)** BiFC interaction assays in *Nicotiana benthamiana* pavement cells transiently co-expressing N-terminal yellow fluorescent protein (nYFP)-SOG1 and C-terminal YFP (cYFP)-IN10/11. Panels **(C,D)** are in normal conditions (treatment with DEX and 2 μg/ml DMSO), and **(E,F)** are treated with DEX and 20 μg/ml antimycin A. Scale bars: 50 μm. **(C–F)** Labels above each panel indicate the filter channel imaged. DIC, differential interference contrast images (left side of each panel); YFP, fluorescence images (middle of each panel); and Merge, the DIC and YFP images combined (right side of each panel). SOG1-nYFP/KIN10-cYFP, co-expression of 35S::SOG1-nYFP and 35S:: KIN10-cYFP; SOG1-nYFP/KIN11-cYFP, co-expression of 35S::SOG1-nYFP and 35S::KIN11-cYFP. **(E,F)** Red arrows indicate nucleus. **(G)** *In vivo* interaction of SOG1 with KIN10/11. DMSO acts as a control. Black arrow, SOG1-Myc. IP, immunoprecipitation; WB, Western blotting.

Since mutations of *KIN10, KIN11*, and *SOG1* suppressed the seedling-lethal phenotype of *sd3* and also restored the short-hypocotyl phenotype in darkness, we speculated that KIN10, KIN11, and SOG1 physically interact to exert their effect. To test whether SOG1 interacts with KIN10 and KIN11, yeast two hybrid assays (Y2H) were performed. Co-expression of both combinations of SOG1 baits with KIN10 or KIN11 prey, and KIN10 or KIN11 baits with SOG1 prey resulted in yeast growth on selective plates (Figure 5A). These results demonstrate that SOG1 and KIN10 or KIN11 physically interact *in vivo*. To confirm this interaction *in planta*, bi-molecular fluorescence complementation (BiFC) assays using transient expression in *Nicotiana benthamiana* were performed. The N-terminal half of yellow fluorescent protein (nYFP) was fused to SOG1 (nYFP-SOG1) and the C-terminal half of YFP was fused to KIN10 or KIN11 (cYFP-KIN10 and cYFP-KIN11, respectively), both under a DEX-inducible promoter (Figure 5B). As shown in Figure 5C,D, co-transformation of either nYFP-SOG1 and cYFP-KIN10, or nYFP-SOG1 and cYFP-KIN11 constructs did not produce any YFP fluorescence after DEX treatment. However, after treatment of leaves with antimycin A, YFP fluorescence was observed in the nuclei of epidermal cells (Figure 5E,F).

Subsequently, to confirm the Y2H and BiFC results, we carried out a pull-down assay. Myc-tagged SOG1 (SOG1-Myc) under its own promoter was stably expressed in *Arabidopsis thaliana* (*pSOG1*::*SOG1*-*Myc*). After immunoprecipitation with an anti-AMPKα subunit antibody, Western blot analysis detected a band corresponding to SOG1-Myc fusion protein only in transgenic plants treated with antimycin A (Figure 5G).

### SOG1 Is Phosphorylated Under Low Amount of Intracellular ATP

We further analyzed whether SOG1 was modified by phosphorylation. It was predicted that SOG1 has several phosphorylation sites (Yoshiyama et al., 2013). Considering that SOG1 interacts with KIN10 or KIN11 *in vivo* (Figure 5), we speculated that these kinases phosphorylate SOG1.

**FIGURE 6:**
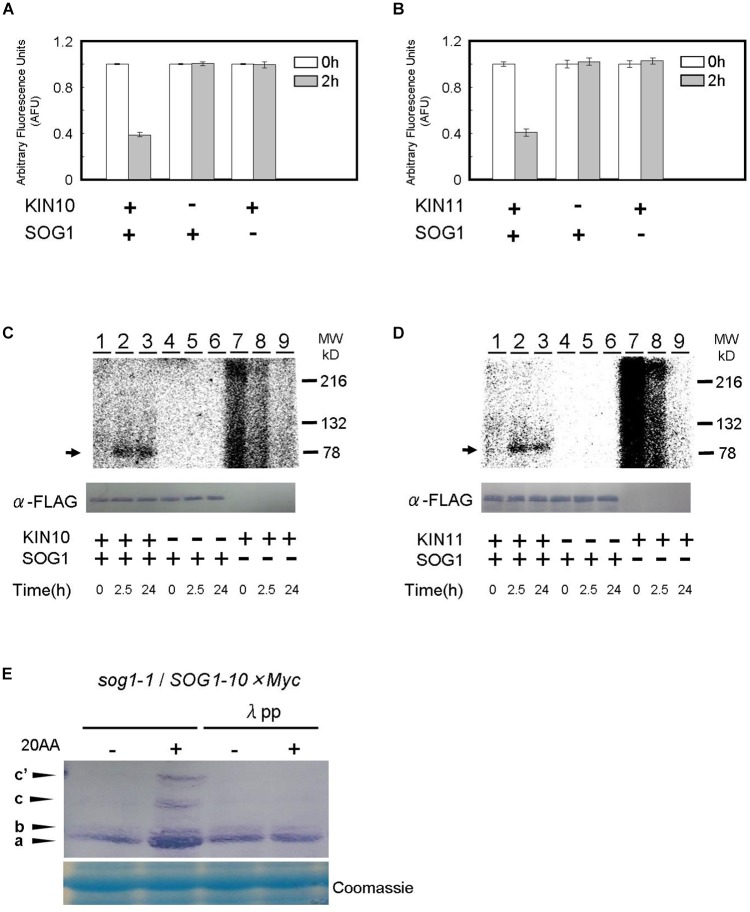
SOG1 is directly phosphorylated by SnRK1 in low ATP. **(A,B)** The SOG1 peptide was used as the kinase substrate in a Kinase-Glo^TM^ Luminescent assay. The reduction in ATP levels in the reaction mixture indicates the degree of phosphorylation by the **(A)** KIN10 or **(B)** KIN11 kinase. Error bars indicate standard error of three biological replicates. **(C,D)** *In vitro* kinase assays with Flag-tag-KIN10 **(C)** or Flag-tag-KIN11 **(D)** and Flag-tag-SOG1 as a substrate. Immunoblotting with the anti-FLAG antibody shows equal FLAG-tagged SOG1 protein was loaded (bottom panel). **(E)** SOG1-Myc transgenic plants were treated with DMSO or 20 μM of antimycin A (20AA). Total protein was separated by a SDS-PAGE gel with Phos-tag and used for immunoblotting with the anti-Myc antibody. In addition to the main band (a), slow migrating bands (b, c, c’) can be observed. From left; protein blot from plants treated with DMSO and 20AA; DMSO with λ protein phosphatase (λPP) treatment and 20AA with λPP treatment. CBB, Coomassie Brilliant Blue, staining shows equal loading.

We firstly used a Kinase-Glo^®^ luminescent kinase assay in which reduction of the amount of ATP in the reaction represents the consumption of ATP and the occurrence of phosphorylation (see “Materials and Methods” section). Recombinant KIN10, KIN11, and SOG1 proteins were synthesized in a cell-free system using wheat germ extract. When the KIN10 or KIN11 proteins were add to the reaction containing SOG1 protein as a substrate, the amount of ATP decreased to approximately one half after a 2 h incubation compared to a 0 h incubation. On the other hand, ATP levels did not decrease when SOG1 or KIN10/11 was not present in the reaction (Figure 6A,B). These data indicate that SOG1 is directly phosphorylated by KIN10 and KIN11 *in vitro*.

To confirm the Kinase-Glo^®^ luminescent kinase assays, SOG1 protein was incubated for 2.5 h and 24 h, respectively, in the presence of [γ^32^P]ATP and phosphorylation was monitored by autoradiography after SDS-PAGE. SOG1 phosphorylation was detected in both the 2.5 h and 24 h incubated reactions containing KIN10 (Figure 6C, lanes 2–3) and KIN11 (Figure 6D, lanes 2–3). We did not observe SOG1 phosphorylation when KIN10 (Figure 6C, lanes 5–6) or KIN11 (Figure 6D, lanes 5–6) was not added. We did not observe any specific bands when SOG1 was absent (Figure 6C,D, lanes 7–9, respectively). We observed unknown high background in both KIN10 and KIN11 at 0 h incubation which decreased with incubation time (Figure 6C,D, lanes 7–9). Taken together with the evidence in Figure 6A,B, these results demonstrate that SOG1 is directly phosphorylated by KIN10 and KIN11 *in vitro*.

We further analyzed whether SOG1 is phosphorylated in response to a low amount of ATP *in vivo*. Plants overexpressing *SOG1-Myc* were grown on medium for 9 days, and for 1 day after treatment with or without antimycin A in continuous light. Total protein was extracted, separated by SDS-PAGE with or without Phos-tag and immunoblotted with a Myc-specific antibody for detecting SOG1-Myc. Phos-tag is a phosphate chelator and phosphorylated proteins migrate slower than unphosphorylated ones. As shown in Figure 6E, immunoblots of proteins extracted from antimycin A-treated plants showed slower migrating bands (c and c’) in addition to the 80-kDa protein (band a). To confirm whether these bands shift depending on the phosphorylation of SOG1, the extracted proteins were incubated with λ protein phosphatase before SDS-PAGE with Phos-tag, with the result that the slower migrating bands were not detected (Figure 6E). These data suggest that phosphorylation of SOG1 occurs after treatment with antimycin A.

### Expression of Genes Involved in Negative Regulation of Endocycle Are Up-Regulated in *sd3*

**FIGURE 7:**
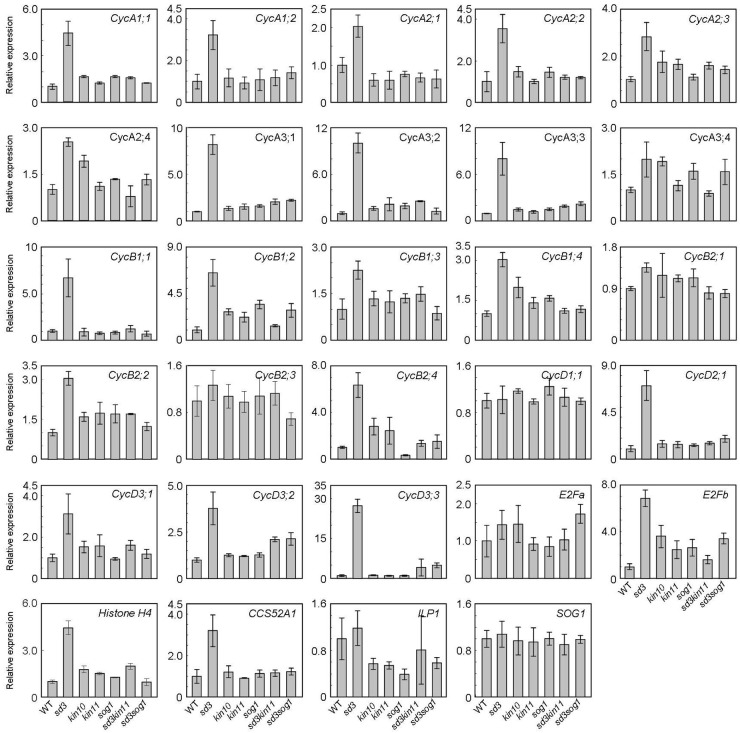
Expression levels of cyclin genes in WT and mutants. Total RNA prepared from 5-day-old dark-grown seedlings (approximately 20) was used to amplify each gene by RT-PCR. All values were normalized against the expression of the *AtACTIN2* gene. Error bars indicate standard deviation of three biological replicates.

To understand the short-hypocotyl phenotype caused by mitochondrial mutation, we examined expression of cell cycle-related genes in the *sd3* mutation. *CYCA2s*, especially *CYCA2;3* and *CYCA2;1*, are suggested to act in the transition from the mitotic cycle to the endocycle that enables cell elongation in the hypocotyl (Imai et al., 2006; Yoshizumi et al., 2006). Hence, CYCA2s are suggested to be the negative regulators for progression of the endocycle. Expression of these genes was up-regulated in the *sd3* mutant in darkness (Figure 7), although *ILP1*, a repressor of *CYCA2s* transcription, was not changed. Additionally, *CYCA1s*, and *CYCA3s* (except *CYCA3;4s*) were also up-regulated (Figure 7). *CYCD3s* (especially *CycD3;1* and *CYCD3;3*), suggested to act in the transition between promoting mitotic cell division and inhibiting the endocycle (Dewitte et al., 2003, 2007; Menges et al., 2006) were also up-regulated. In the *sd3kin11* and *sd3sog1* mutants, expression levels of these genes were same as WT. However, expression of *CYCD1;1, CYCB2;1, CYCB2;3*, and *E2Fa*, which enhance the mitotic cell cycle, was not different between WT and the mutants.

Considering the differential expression of some genes in the different genetic backgrounds described above, there are questions about the transduction of the low ATP signal from mitochondria to the nucleus. This type of communication between the organelles is called a mitochondrial retrograde signal (MRS). *AOX1a* expression is known as a marker for active MRSs (Saisho et al., 2001; Dojcinovic et al., 2005; Zarkovic et al., 2005). To elucidate whether the *sd3* mutation connects with the induction of a MRS, we measured the expression of *AOX1a* by qRT-PCR. *AOX1a* expression is 45 times higher in *sd3* than in WT (Supplementary Figure S4). In *kin10, kin11, sog1* single and *sd3sog1* double mutants, the expression is the same as WT and in *sd3kin11* it is also much lower than that of *sd3* but higher than WT.

## Discussion

From the evidence in this study, we propose that intracellular ATP can be a signal for plant growth as well as an energy source. We describe plant mitochondria dysfunction and how a low amount of ATP affects cell cycle progression. Previously, Guérinier et al. (2013) reported that SnRK1 controls cell proliferation through phosphorylation of KRP6, the Arabidopsis CDK inhibitor p27kip1 homolog. There is only a small subset of information that addresses how the amount of intracellular ATP controls cell elongation and cell division in plants. We found that mitochondrial dysfunction is suppressed by the *SnRK1* and *SOG1* mutations, and that the corresponding proteins are possible components of the signaling pathway.

### Mutations in α-Subunits of SnRK1 and SOG1 Restore Mitochondrial Dysfunction and Influence Cell Elongation and Differentiation

Our previous work reported that compound C, an inhibitor of AMPK, suppresses inhibition of hypocotyl elongation in the *tim50* mutant (Kumar et al., 2012). In addition, our preliminary data showed that compound C suppresses inhibition of hypocotyl

elongation in the *sd3* mutant and antimycin A treated seedling. Suppression similar to that by compound C was also seen in the *sd3kin10* and *sd3kin11* double mutants (Figure 2). In both cases, the seedling-lethal and short-hypocotyl phenotypes of *sd3* were suppressed by adding another mutation either in KIN10 or KIN11, the α-subunits of SnRK1. Although, KIN10 and KIN11 have same function, *sd3kin10* showed much smaller at their adult stage, comparison with *sd3kin11* (Supplementary Figure S3). These growth difference would come from different expression profiles between *KIN10* and *KIN11* (Zimmemann et al., 2005; Fragoso et al., 2009).

*sog1* mutants are more resistant to hypocotyl inhibition caused by antimycin A (Figure 3B) and maintain their higher polyploidy fractions (Figure 3C,D), indicating SOG1’s role in signaling from the mitochondria. This suppression by antimycin A was further confirmed in the *sd3sog1* double mutants, and the *sd3* seedling-lethal and short-hypocotyl phenotypes are partially rescued by the *sog1* mutation in a similar manner to the *kin11-1* mutation (Figure 3E). In darkness, both the *sd3kin11* and *sd3sog1* double mutants overcome the short-hypocotyl phenotype of *sd3* (Figure 4A,B). However, the *sog1* mutation does not effectively rescue the *sd3* mutant compared to the *kin11* mutation. Although *SOG1* is a single gene in the *Arabidopsis* genome there may be another functionally similar gene that controls hypocotyl elongation.

Importantly, the abnormal mitochondria in *sd3* are not restored by the *sog1* or *kin11* mutations (Figure 4E), and *sd3kin11* and *sd3sog1* show much lower intracellular ATP levels as compared to *sd3* in the dark (Figure 4D). These results suggest that the hypocotyl elongation caused by the cell elongation observed in *sd3kin11* and *sd3sog1* is not caused by a recovery in the amount of intracellular ATP and that a low ATP (or high AMP) signal initiated from the mitochondria to arrest hypocotyl elongation can be blocked by mutations in the SnRK1 α-subunits and SOG1. It is possible that intracellular ATP is not only an energy source but also acts as a signal for the control of the endocycle in hypocotyl elongation.

### SOG1 Interacts With KIN10 and KIN11 as a Switch for a Signal Triggered by the Intracellular ATP Level

We observed that SOG1 interacts with KIN10 and KIN11 *in vitro* and *in vivo*. This interaction was seen only in the presence of antimycin A *in vivo* using *N. benthamiana* leaf epidermal cells and *Arabidopsis thaliana* transgenic plants (Figure 5C–G). A low ATP signal from mitochondria may trigger the interaction of KIN10 and KIN11 with SOG1 for the control of cell elongation and cell division. AMPK is known to be activated by a certain AMP/ATP ratio accompanied by a conformation change and phosphorylation (Wilson et al., 1996; Polge and Thomas, 2007) and the activated AMPK interacts with the substrates. In mammals, AMPK interacts with p53, a transcription factor for the suppression of tumors, to be modified by phosphorylation when intracellular glucose is low (Okoshi et al., 2008). In a similar way, a low amount of ATP caused by antimycin A treatment triggered interaction between SOG1 and SnRK1 (KIN10/11) and phosphorylation of SOG1 (Figure 5, 6), although it has not been demonstrated that KIN10/11 directly phosphorylate SOG1 *in vivo*. A recent report also showed that transcription factor bZIP11 interacts with SnRK1 when the ATP level is low during root growth (Weiste et al., 2017). As a response to low energy, SnRK1 directly interacts with bZIP63, a regulator of the starvation response (Mair et al., 2015). And, that bZIP63 controls expression of mitochondrial genes in the low-energy response (Pedrotti et al., 2018).

In our previous publication, we indicated that antimycin A treatment seedlings is caused reduction of ATP level (Hamasaki et al., 2012). It was reported that reduction of ATP caused phosphorylation of SnRK1 (Broeckx et al., 2016). Also, SnRK1 kinase is activated when treatment with AMP in spinach (Sugden et al., 1999). We concluded that phosphorylation of SnRK1 subunit (KIN10 and KIN11) would be promoted interaction to SOG1 in reduction of ATP level caused by antimycin A. Hence, it is speculated that interaction between SnRK1 and its transcription factors and their phosphorylation are important steps for recognition of a low amount of ATP and the subsequent reaction of the plant.

It is known that SOG1 is phosphorylated by AtATM, a major regulator of DNA double strand breaks induced by UV or H_2_O_2_ (Yoshiyama et al., 2013; Yi et al., 2014), and also by AtATR, closely related to AtATM, which monitors for DNA damage induced by high aluminum (Al) accumulation in root tip cells (Sjogren et al., 2015). Thus, control of SOG1 by phosphorylation may be the key controlling point for plant survival. In SOG1, several phosphorylation sites have been predicted and SOG1’s direct target gene expression level is dependent on the number of phosphorylation sites of SOG1 (Yoshiyama et al., 2017). Further study will be needed to elucidate which phosphorylated site is important for SnRK1 during low ATP signaling.

### SnRK1 and SOG1 Are Components of a Novel Mitochondrial Retrograde Signaling (MRS) Pathway Mediating the Low Energy Response Triggered by Low ATP Levels

Several papers have reported MRSs where signal transduction from the mitochondria to the nucleus occurs to control nuclear gene expression. The amount of the ATP and reactive oxygen species (ROS) produced by cellular processes and stress are dependent on mitochondrial function. Hence, ATP and ROS are considered as molecular factors responsible for signal transduction in MRSs (Butow and Avadhani, 2004; Owusu-Ansah et al., 2008; Kleine and Leister, 2016). This kind of regulation has also been reported in *Drosophila.* A knockout mutation of the mitochondrial cytochrome c oxidase subunit (CoVa) blocks the G1/S transition during the mitotic cell cycle (Mandal et al., 2005, 2010; Owusu-Ansah et al., 2008; Millau et al., 2009). They reported that there is a signaling pathway triggered by a low amount of intracellular ATP caused by dysfunction of mitochondria and that AMPK, p53 and other signaling molecules including cyclin E, a negative regulator of the G1/S transition, are components in this MRS pathway.

In plants, it is well-known that chloroplast function is coupled with the nucleus to enable efficient photosynthesis and that genomes uncoupled (*gun*) mutants were isolated as being deficient in the communication between organelle and nucleus (Susek et al., 1993). In the case of mitochondria, it is known that some MRSs are triggered by an increase in ROS, which is caused by abiotic stress (Dojcinovic et al., 2005; Zarkovic et al., 2005). However, there is little information regarding MRSs caused by low amounts of mitochondrial ATP in plants. We have shown that expression of *AOX1a*, a marker for an active MRS, is much higher in *sd3*, where mitochondria are partly disrupted, than in WT, *sd3sog1* and *sd3kin11* (Supplementary Figure S4). However, expression of some cell cycle-related genes is higher in *sd3* mutants but suppressed in the double mutants (Figure 7). These results provide evidence of the communication between mitochondria and the nucleus. We suggest that this signaling pathway, initiated by a low amount of ATP caused by mitochondrial disruption, is a novel retrograde signal in plants.

### Possible Mechanism of Intracellular ATP for the Control of Plant Growth

**FIGURE 8:**
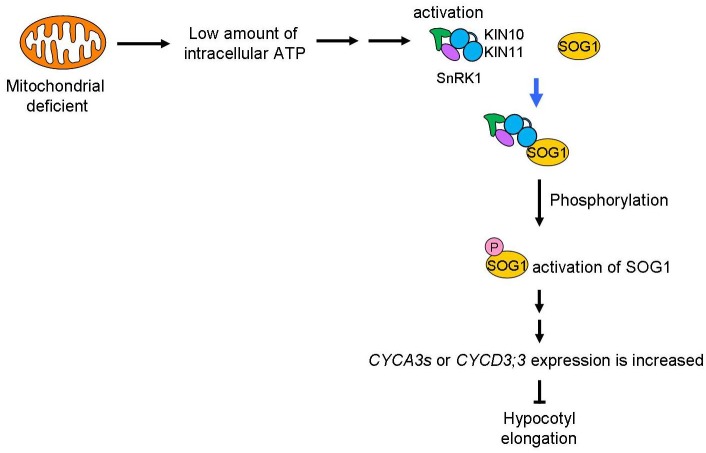
Mitochondrial signal for the control of plant growth. Mitochondrial activity is transduced through SnRK1 and SOG1 to control plant growth. A low level of ATP caused by dysfunction or inhibition of the mitochondria will be sensed by SnRK1 and trigger interaction with SOG1, a NAC-type transcription factor. This “ATP switch” will then cause phosphorylation of the SOG1 protein leading to its activation. Next, expression of cell-cycle related genes (*CYCA3*s or *CYCD3*;3) are increased to suppress plant cell growth. Therefore, as a result, low mitochondrial activity causes suppression of plant cell growth using ATP as the signal.

A schematic of the signaling pathway, triggered by low amounts of intracellular ATP caused by mitochondrial disruption that arrests the cell cycle, is shown in Figure 8. We speculate that deficient mitochondrial activity results in low amounts of intracellular ATP. In this study, we do not have enough information about how low amount of ATP levels affects SnRK1 activation. SnAKs (SnRK1 activated kinase) exist in plant specifically and these kinase would have function as AMP sensor (Crozet et al., 2010). Further study will be needed to elucidate whether SnAKs act in this signal transduction. The transduction of the low ATP signal would reach to SnRK1 and its α-subunits, KIN10/11, directly interact with SOG1 in these conditions and so function as an ATP switch. SOG1 is phosphorylated in low ATP concentrations and this phosphorylation may be triggered by SnRK1. Finally, expression of cell cycle-related genes, such as the *CYCA2s* or *CYCD3s* involved in repression of endocycle progression, is altered. At this time, expression of cell proliferation-related genes, *CYCB1;1, CYCB1;2*, and *CYCB2;4*, was also higher in *sd3*. It is known that *CYCA2s* are repressors of the endocycle (Imai et al., 2006). However, expression of *ILP1*, a repressor of *CYCA2s*, and *CYCA2s* in *sd3* are not clearly different from those in WT, while expression of *CYCDs* are different between the two (Figure 7). These data suggest that ILP1 and CYCA2s are not therefore involved in inhibiting the endocycle in response to a low amount of ATP. *CYCA3s* are highly accumulated in *sd3*. A previous report showed that plants overexpressing *Nicta;CycA3;2*, which encodes A-type cyclin of tobacco, exhibited a decrease in the endocycle (Yu et al., 2003). Additionally, it is known that *CYCA3s* (except *CYCA3;3*) are expressed during S phase (Menges et al., 2005; Van Leene et al., 2010), suggesting that *CYCA3s* negatively regulate the endocycle.

It is reported that *CYCD3;1* is increased during depressed levels of sugar (Menges et al., 2006). Therefore, *CYCDs* may play an important role in modulating plant growth development in response to the amount of intracellular ATP. More recently, SOG1-binding sequence have been identified and several genes, including cell cycle related gene, are isolated as direct targets of SOG1 (Weimer et al., 2016; Ogita et al., 2018). Further studies will elucidate a more complete picture of the signaling mechanisms from the mitochondria that influence the cell cycle and plant growth.

## Materials and Methods

### Plant Material and Growth Conditions

*Arabidopsis thaliana* plants were grown under long-day conditions (16 h light and 8 h darkness) at 22°C on GM (Germination Medium) agar plates (Valvekens et al., 1998). *Arabidopsis thaliana* accessions Columbia-0 (Col-0) and Nossen-0 (No-0) were used as wild type. The *sd3* mutants, in the No-0 background, have been described previously (Hamasaki et al., 2012). The *kin10* (CS455545) and *kin11* mutants (CS851107), in Col-0, were obtained from the T-DNA insertion mutant collection of the Salk Institute Genome Analysis Laboratory (Alonso et al., 2003). To screen for homozygous lines of the *kin10* and *kin11* mutants, the following primer pairs were designed: 5′-ATATTGACCATCATACTCATTGC-3′ and 5′-AAAATCAATCTTGGTGGCATG-3′ for *kin10*; 5′-CCTCTAGTGGTTATCTCGGGG-3′ and 5′-AAAATCAAT CTTGGTGGCATG-3′ for WT, and 5′-TCTGGGTTTTG TGGTTTCTTG-3′ and 5′-ATCCGAGAATCAAAAACCCAG-3′ for *kin11*; 5′-AACGTCCGCAATGTGTTATTAAGTTGTC-3′ and 5′-GAAAACTGACAAGAACCACCG-3′ for WT. mRNA accumulation of each gene in the corresponding mutant was examined by semi-quantitative RT-PCR analysis using specific primer sets (Supplementary Table S1). *sog1*, in the Ler background, was kindly provided by Keiko Sugimoto-Shirasu (RIKEN Center for Sustainable Resource Science). *sog1* was backcrossed three times into the Col-0 background.

Sterilized seeds were plated on GM agar plates (Valvekens et al., 1998). Plants were placed at 4°C in the dark for 4 days and then incubated under long-day conditions (16 h light and 8 h darkness) at 22°C or covered with aluminum after red-light treatment for 6 h. Seedlings were grown on plates placed horizontally.

For chemical treatments, antimycin A and compound C were dissolved in DMSO as stock solutions of 10 mM and 20 mM, respectively, and diluted into GM agar plates. DEX was dissolved in ethanol as a stock solution of 20 mM, and diluted into GM agar plates. Control experiments were treated with the corresponding quantity of DMSO or ethanol alone.

### Plant Transformation Constructs

For Y2H and BiFC (split YFP) studies, the full-length open reading frames of *SOG1, KIN10*, and *KIN11* (without their stop codons) were amplified from an Arabidopsis cDNA library (Life Technologies) by PCR with SOG1-F 5′-GGGGACAAGTTTGTACAAAAAAGCAGGCTTCATGGCTG GGCGATCATGGCTGATCG-3′ and SOG1-R 5′-GGGGA CCACTTTGTACAAGAAAGCTGGGTCATCAGTCTTTCCAG TCCCCCAAG-3′ primers for *SOG1*, KIN10-F 5′-GGGG ACAAGTTTGTACAAAAAAGCAGGCTTCATGTTCAAACGA GTAGATGAG-3′ and KIN10-R 5′-GGGGACCACTTTG TACAAGAAAGCTGGGTCGAGGACTCGGAGCTGAGCAAG AAAAGC-3′ primers for *KIN10*, KIN11-F 5′-GGGGACAAGTTTGTACAAAAAAGCAGGCTTCATGGATC ATTCATCAAATAGATTTGGC-3′ and KIN11-R 5′-GGGGACCACTTTGTACAAGAAAGCTGGGTCGATCACAC GAAGCTCTGTAAG-3′ primers for *kin11*, and cloned into the pDONR207 ENTRY vector by BP recombination according to the manufacturer’s instructions (Invitrogen Corp.). For Y2H studies, these open reading frames were transformed into the *pGAD*-*RC* (prey vector) or *pGBK*-*RC* (bait vector) destination vectors (Ito et al., 2000) to fuse in frame with the Gal4-binding domain (Gal4-BD) or Gal4-activation domain (Gal4-AD), respectively, by LR reaction to create the bait and prey. For BiFC studies, these open reading frames were transformed into the p7002YN^GW^ or p7002YC^GW^ destination vectors to be under the control of the DEX-inducible promoter and fused to the N- or C-terminus of YFP (yellow fluorescence protein) by LR recombination. These destination vectors have been described previously (Fujioka et al., 2007). Each construct was transformed into *Agrobacterium* GV3101.

### Flow Cytometric Analysis

Extraction and staining for polyploidy were performed by CyStain^®^ UV Ploidy, according to the manufacturer’s instructions (Partec, Münster, Germany). Flow cytometric analysis was performed as described previously (Hamasaki et al., 2012).

### Quantitative PCR Analysis

RNA was extracted from 2-week-old *Arabidopsis* seedlings using TRIzol reagent (Invitrogen, Carlsbad, CA, United States). First-strand cDNA was prepared from total RNA using the Superscript^TM^ First-Strand Synthesis System (Invitrogen, Carlsbad, CA, United States), according to the manufacturer’s instructions. For quantitative PCR, a SYBR Green Real-Time PCR Master Mix (TOYOBO, Tokyo, Japan) was used. Reactions were run and analyzed on the MX3000P Multiplex Quantitative PCR System (Promega Corp.), according to the manufacturer’s instructions. Quantitative reactions were done in triplicate and averaged. The primers used are described in Supplementary Table S1.

### ATP Measurement

Extraction and measurement of intercellular ATP were performed as described previously (Hamasaki et al., 2012). Five-day-old dark-grown seedlings were used. Twenty seedlings were boiled with 300 μl of sterilized water for 15 min. Extracted ATP is measured using ATP determination kit (Molecular Probes^TM^). The luminescence of samples was measured using a Turner Designs TD20/20 Luminometer (Sunnyvale, CA, United States).

### Yeast Two-Hybrid Assay

Transformation and mating of yeasts were performed as described previously (Kuroda et al., 2002). *Saccharomyces cerevisiae* strains Y182 (*MAT*α, *ura3-52, his3-200, ade2-101, trp1-901, leu2-3, 112, gal4*Δ, *met–, gal80*Δ, *MEL1, URA3*::*GAL1UAS*-*GAL1TATA*-*lacZ*) and AH109 (*MATa, trp1-901, leu2-3, 112, ura3-52, his3-200, gal4*Δ, *gal80*Δ, *LYS2::GAL1UAS-GAL1TATA-HIS3, GAL2UAS-GAL2TATA-ADE2, URA3::MEL1UAS-MEL1TATA-lacZ, MEL1*), and the *pGAD*-*RC* and *pGBK*-*RC* vectors were used for the yeast two-hybrid assays. Selection was performed on SC (Synthetic Complete) plates without leucine, tryptophan and histidine.

### Bi-Molecular Fluorescent Complementation

The constructs for the BiFC assays were transformed into the *Agrobacterium tumefaciens* GV3101 strain harboring plasmid pMP90. These agrobacteria were mixed and infiltrated into the leaf epidermal cells of *N. benthamiana*. *Nicotiana benthamiana* plants were agro-infiltrated according to Lewis et al. (2008). After 48 h, leaf discs were put in liquid MS medium containing DEX and antimycin A and a vacuum was applied for 10 min. After vacuum, the samples were incubated at room temperature for 6 h to overnight and observed by microscopy. Fluorescence was observed using an Axiovert 200M microscope (Zeiss, Carl, Germany) equipped with a LSM 510/510 META confocal scanner (Zeiss, Carl, Germany).

### Electron Microscopy

WT, *sd3, kin11, sog1, sd3kin11*, and *sd3sog1* mutant lines were grown in darkness for 5 days and hypocotyls from each were prefixed in 2% glutaraldehyde and 4% paraformaldehyde in 50 mM sodium cacodylate buffer at 4°C for 4 h, and then postfixed with 2% osmium tetroxide in a 50 mM sodium cacodylate buffer at 4°C for 2 h. Fixed samples were dehydrated in an ethanol series, embedded in Spurr resin and polymerized at 72°C. Ultra-thin sections were prepared with a diamond knife, and stained with uranyl acetate and lead citrate. Observations were made on a JEM-1400 transmission electron microscope (JEOL, Tokyo, Japan).

### SOG1 Phosphorylation Assay

The transgenic plants expressing the SOG1-Myc gene under the control of SOG1’s native promoter (WT/pSOG1::SOG1-Myc) (Yoshiyama et al., 2013) were grown under long-day conditions (16 h light and 8 h darkness) at 22°C on GM agar plates (Valvekens et al., 1998). Fourteen-day-old plants were transferred into liquid MS medium containing DMSO or antimycin A and incubated for 3 days. Total protein was extracted from 100 mg of whole seedlings using the following buffer: 50 mM Tris-HCl (pH 8.0), 150 mM NaCl, 0.1% 2-mercaptoethanol, 5% glycerol, 0.5% Triton X-100, and cOmplete^TM^ protease inhibitor mini cocktail (Sigma). The slurry was centrifuged twice to remove precipitates, and 20 μl of the SDS denaturated supernatant were loaded onto a 7.5% SDS-polyacrylamide gel for electrophoresis. To detect phosphorylated SOG1 proteins, Phos-tag reagent was used in a phosphor protein mobility shift assay to detect phosphorylated SOG1 protein. After electrophoresis, the proteins were electroblotted to a polyvinylidene difluoride (PVDF) membrane (Millipore) in the following buffer: 25 mM Tris, 192 mM glycine and 20% methanol. Subsequently, the membrane was incubated for 2.5 h in skimmed milk, rinsed two times with 1 × TBST, and immunoblotted using mouse anti-Myc antibody (Nacalai Corp., 1:1000 dilution) for 12 h. After incubation, the membrane was rinsed three times and incubated for 1.5 h with an anti-mouse immunoglobulin alkaline phosphatase secondary antibody (Promega Corp., 1:5000 dilution) to detect SOG1-Myc. The membrane was then rinsed three times with 1 × TBST and incubated with Western Blue^®^ Stabilized Substrate for Alkaline Phosphatase (Promega Corp.).

### Pull-Down Assay

Proteins extracted from 500 mg of whole seedlings were incubated for 3 h with AMPKα antibody. Subsequently, Protein G Sephalose (GE Healthcare) was added to the extracts that were then shaken for 1.5 h at 4°C. The beads were rinsed four times with extraction buffer, and then the bound proteins were boiled with SDS sample buffer. The immunoprecipitated proteins were detected by immunoblotting using a mouse anti-Myc antibody and an anti-mouse immunoglobulin alkaline phosphatase secondary antibody.

### Kinase Assay

The cDNAs of *KIN10* and *KIN11* were amplified by PCR. The primers used were 5′-GGAAACTC GAGATGTTCAAACGAGTAGATGAG-3′ and 5′-CCTTT GCGGCCGCTCAGAGGACTCGGAGCTGAGCAAG-3′ for *KIN10*, 5′-GGAAACTCGAGATGGATCATTCATCAAATAGA TTTGG-3′ and 5′-CCTTTGCGGCCGCTCAGATCACACG AAGCTCTGTAAG-3′ for *KIN11*. These gene were cloned using XhoI/NotI into the equivalent sites of the pEU vector (Cell Free Science). The proteins were expressed in wheat germ extract (WEPRO7240H Expression Kit) using the Protemist-DT II, an automatic machine (Cell Free Science), according to the manufacturer’s instructions.

*In vitro* kinase activity of KIN10 and KIN11 was measured using an ATP detection reagent (Kinase-Glo^®^ Plus Luminescent Kinase Assay; Promega). KIN10 or KIN11, and the SOG1 peptide were incubated for 2 h in kinase reaction buffer [40 mM HEPES (pH 7.5 NaOH), 20 mM MgCl_2_, 4 mM DTT, 100 μM ATP] at room temperature. Reaction mixture without kinase was used as a negative control. After incubation, Kinase-Glo^®^ Reagent was added to the reaction mixture and incubated at room temperature for 20 min. The luminescence of samples was measured using a Turner Designs TD20/20 Luminometer (Sunnyvale, CA, United States). Three biological replicates were used for each sample analyzed.

For the radioactive assay, 4 μCi γ-32P-labeled ATP (PerkinElmer) were added to each reaction. The reactions were purified using Anti-FLAG M2 affinity gels (Sigma). Purified proteins were separated by SDS-PAGE, exposed to an imaging plate (Fujifilm), and the radioactive signals were visualized using a Typhoon FLA7000 (GE Healthcare).

### SOG1 Phosphorylation Assay

The transgenic plants expressing the SOG1-Myc gene under the control of SOG1’s native promoter (WT/pSOG1::SOG1-Myc) (Yoshiyama et al., 2013) were grown under continuous light at 22°C on MGRL (pH 5.6) medium (Fujiwara et al., 1992). Nine-day-old plants were transferred into new MGRL medium containing DMSO or antimycin A and incubated for 24 h. Total protein was extracted from 100 mg of whole seedlings using the following buffer: 50 mM Tris-HCl (pH 8.0), 150 mM NaCl, 0.1% 2-mercaptoethanol, 5% glycerol, 0.5% Triton X-100, and complete^TM^ protease inhibitor mini cocktail (Sigma). The slurry was centrifuged twice to remove precipitates, and 20 μl of the SDS denaturated supernatant were loaded onto a 7.5% SDS-polyacrylamide gel for electrophoresis. To detect phosphorylated SOG1 proteins, Phos-tag reagent (Wako Corp.) was used in a phosphor protein mobility shift assay. After electrophoresis, the gel was rinsed twice with transfer buffer (25 mM Tris, 192 mM glycine and 20% methanol) containing 1 mM EDTA, followed by a rinse with transfer buffer without EDTA. The proteins were semi-dry electroblotted to a PVDF membrane (Millipore) in transfer buffer. Subsequently, the membrane was incubated for 2.5 h in skimmed milk, rinsed two times with 1 × TBST, and immunoblotted using mouse anti-Myc antibody (Nacalai Corp., 1:1000 dilution) for 12 h. After incubation, the membrane was rinsed three times and incubated for 1.5 h with an anti-mouse immunoglobulin alkaline phosphatase secondary antibody (Promega Corp., 1:5000 dilution) to detect SOG1-Myc. The membrane was then rinsed three times with 1 × TBST and incubated with Western Blue^®^ Stabilized Substrate for Alkaline Phosphatase (Promega Corp.). Lambda phosphatase (λPP) (New England BioLabs) was used to confirm the phosphorylated SOG1. Aliquots (38 μl) of protein lysate were mixed with 12 μl of phosphatase reaction buffer (1 × λPP buffer, MgCl_2_, 400 U of λPP, and 100 μM MG132) and incubated at 30°C for 30 min. As a negative control, the phosphatase reaction was carried out with 0.5 μl of Phosphatase Inhibitor Cocktail Solution I.

## Author Contributions

HH, YK, HK, and MM conceived and coordinated the study and wrote the manuscript. HH, HK, and NN performed the experiments. TK, YY, and HS provided technical advises. All authors reviewed the results and approved the final version of the manuscript.

## Conflict of Interest Statement

The authors declare that the research was conducted in the absence of any commercial or financial relationships that could be construed as a potential conflict of interest.

## Supplementary Material

FIGURE S1 Expression levels in kin10 and kin11 mutants. **(A)** Real-time RT-PCR analysis of KIN10 expression in WT and kin10. Total RNA prepared from 7-day-old dark-grown seedlings (approximately 20 seedlings) was amplified by RT-PCR. All values were normalized against expression of the *AtACTIN2* gene. Error bars indicate the mean standard deviation of 3 biological replicates. **(B)** Real-time RT-PCR analysis of KIN11 expression in WT and kin11. Total RNA prepared from 7-day-old dark-grown seedlings (approximately 20 seedlings) was amplified by RT-PCR. All values were normalized against expression of the *AtACTIN2* gene. Error bars indicate the mean standard deviation of 3 biological replicates.

FIGURE S2 Expression levels of KIN10-dex and KIN11-dex in presence of DEX. **(A)** Real-time RT-PCR analysis of KIN10 expression in vector control (VC), KIN10-dex #1, and KIN10-dex #2. Total RNA prepared from 7-day-old dark-grown seedlings (approximately 20 seedlings) was amplified by RT-PCR. All values were normalized against the expression of the *AtACTIN2* gene (AtACT2). Error bars indicate the mean standard error of 3 biological replicates. **(B)** Real-time RT-PCR analysis of KIN11 expression in the vector control (VC), KIN11-dex #1, and KIN11-dex #2. Total RNA prepared from 7-day-old dark-grown seedlings (approximately 20 seedlings) was amplified by RT-PCR. All values were normalized against the expression of the *AtACTIN2* gene. Error bars indicate the mean standard error of 3 biological replicates.

FIGURE S3 *sd3kin10*, *sd3kin11* and *sd3sog1* double mutants grew to adult stage and produced seeds. **(A)** Plant architecture of 80-day-old light-grown WT (left), kin10 mutant (middle) and *sd3kin10* double mutant (right). **(B)** Plant architecture of 80-day-old light-grown WT (left), kin11 mutant (middle) and *sd3kin11* double mutant (right). **(C)** Plant architecture of 80-day-old light-grown WT (left), *sog1* mutant (middle) and *sd3sog1* double mutant (right). **(A–C)** Scale bars: 5.0 cm. **(D)** Flower, sheath, bud and main body of WT and several mutants. Scale bar: 0.2 mm. **(E)** Time to true leaf production in WT and several mutants. **(F)** Bolting time of WT and several mutants. **(E,F)** Approximately 60 seedlings were grown. Error bars indicate standard deviation.

FIGURE S4 Expression of AOXa1 is altered in *sd3*. Total RNA prepared from 5-day-old dark-grown seedlings (approximately 20 seedlings) was amplified by RT-PCR. All values were normalized against expression of the *AtACTIN2* gene. Error bars indicate standard deviation of 3 biological replicates.

TABLE S1 Accession number of genes and primer pairs used for qRT-PCR.
