# Supplementary figures and images for: SnRK1 Kinase and the NAC Transcription Factor SOG1 Are Components of a Novel Signaling Pathway Mediating the Low Energy Response Triggered by ATP Depletion
Source: Front Plant Sci. 2019 May 10;10:503. doi: 10.3389/fpls.2019.00503 (PMC6523062; doi:10.3389/fpls.2019.00503)

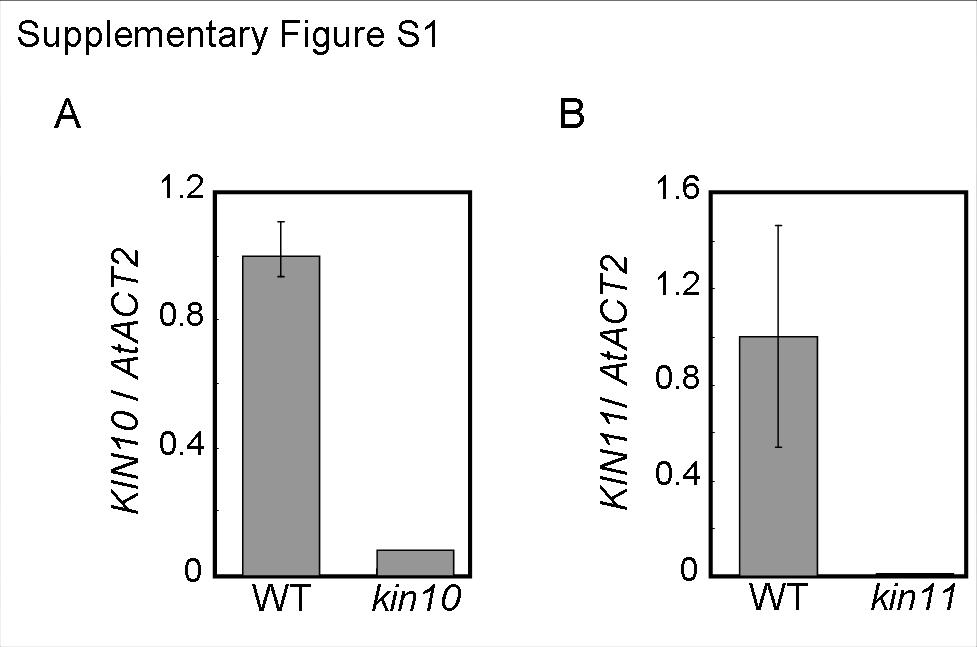

Supplement: FIGURE S1 — Expression levels in kin10 and kin11 mutants. (A) Real-time RT-PCR analysis of KIN10 expression in WT and kin10. Total RNA prepared from 7-day-old dark-grown seedlings (approximately 20 seedlings) was amplified by RT-PCR. All values were normalized against expression of the AtACTIN2 gene. Error bars indicate the mean standard deviation of 3 biological replicates. (B) Real-time RT-PCR analysis of KIN11 expression in WT and kin11. Total RNA prepared from 7-day-old dark-grown seedlings (approximately 20 seedlings) was amplified by RT-PCR. All values were normalized against expression of the AtACTIN2 gene. Error bars indicate the mean standard deviation of 3 biological replicates. [file Image_1.jpg]

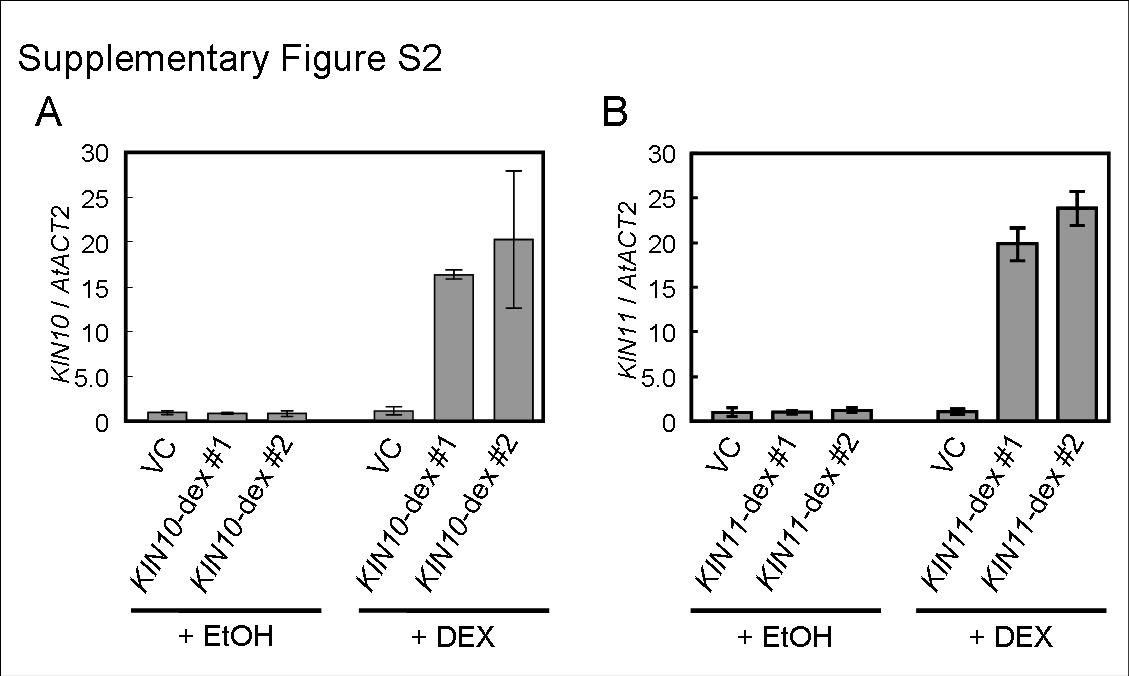

Supplement: FIGURE S2 — Expression levels of KIN10-dex and KIN11-dex in presence of DEX. (A) Real-time RT-PCR analysis of KIN10 expression in vector control (VC), KIN10-dex #1, and KIN10-dex #2. Total RNA prepared from 7-day-old dark-grown seedlings (approximately 20 seedlings) was amplified by RT-PCR. All values were normalized against the expression of the AtACTIN2 gene (AtACT2). Error bars indicate the mean standard error of 3 biological replicates. (B) Real-time RT-PCR analysis of KIN11 expression in the vector control (VC), KIN11-dex #1, and KIN11-dex #2. Total RNA prepared from 7-day-old dark-grown seedlings (approximately 20 seedlings) was amplified by RT-PCR. All values were normalized against the expression of the AtACTIN2 gene. Error bars indicate the mean standard error of 3 biological replicates. [file Image_2.jpg]

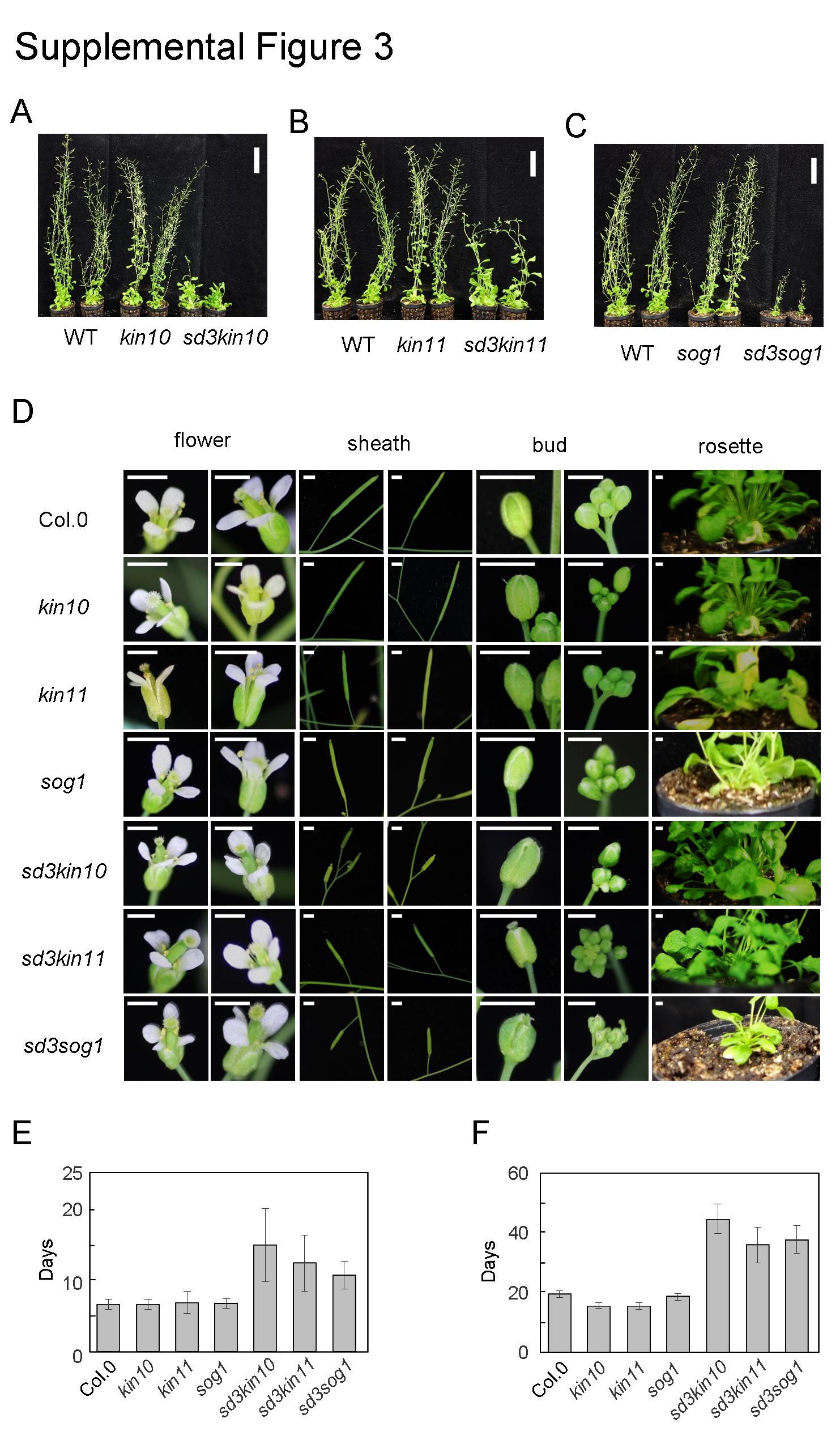

Supplement: FIGURE S3 — sd3kin10, sd3kin11 and sd3sog1 double mutants grew to adult stage and produced seeds. (A) Plant architecture of 80-day-old light-grown WT (left), kin10 mutant (middle) and sd3kin10 double mutant (right). (B) Plant architecture of 80-day-old light-grown WT (left), kin11 mutant (middle) and sd3kin11 double mutant (right). (C) Plant architecture of 80-day-old light-grown WT (left), sog1 mutant (middle) and sd3sog1 double mutant (right). (A–C) Scale bars: 5.0 cm. (D) Flower, sheath, bud and main body of WT and several mutants. Scale bar: 0.2 mm. (E) Time to true leaf production in WT and several mutants. (F) Bolting time of WT and several mutants. (E,F) Approximately 60 seedlings were grown. Error bars indicate standard deviation. [file Image_3.jpg]

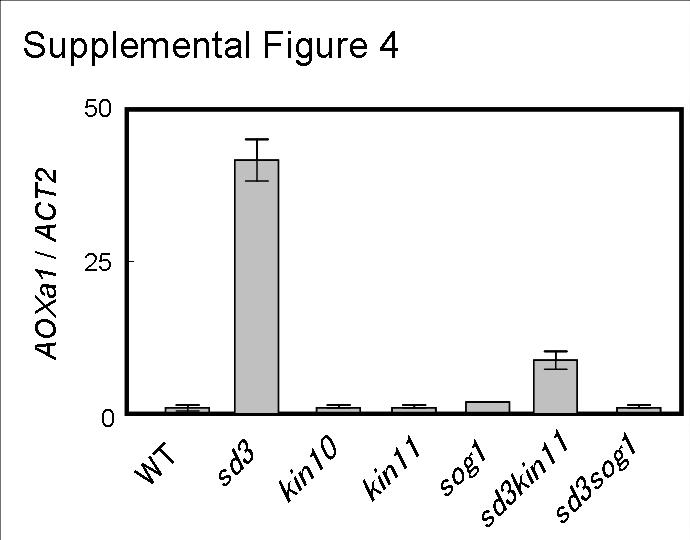

Supplement: FIGURE S4 — Expression of AOXa1 is altered in sd3. Total RNA prepared from 5-day-old dark-grown seedlings (approximately 20 seedlings) was amplified by RT-PCR. All values were normalized against expression of the AtACTIN2 gene. Error bars indicate standard deviation of 3 biological replicates. [file Image_4.jpg]
