# Supplementary material for: SnRK1 Kinase and the NAC Transcription Factor SOG1 Are Components of a Novel Signaling Pathway Mediating the Low Energy Response Triggered by ATP Depletion
Source: Front Plant Sci. 2019 May 10;10:503. doi: 10.3389/fpls.2019.00503 (PMC6523062; doi:10.3389/fpls.2019.00503)
Supplement: TABLE S1 — Accession number of genes and primer pairs used for qRT-PCR. [file Table_1.docx]

Supplemental Table S1

Accession Number of Genes and Primer Pairs Used for qRT-PCR

| Gene Identifier | Genes | Primer Pair |
| --- | --- | --- |
| At3g01090 | KIN10 | 5’-AATCGCTCCGCAACCGAACC-3’ |
|  |  | 5’-TTTCACGGGGATGAGCCCGA-3’ |
| At3g29160 | KIN11 | 5’-ATCCGTCAACACCGTTGGTTCCA-3’ |
|  |  | 5’- CCCGCTTCAGGTGTGCGCAT-3’ |
| AT1g44110 | CYCA1;1 | 5’-AACTGGCATTAACGCAAACAC-3’ |
|  |  | 5’-TCCTCTTCGACGAAATCTTCA-3’ |
| At1g77390 | CYCA1;2 | 5’-CTTGTGGAGGTTGCTGAAGAG-3’ |
|  |  | 5’-TCATCATGCAGGTAACACCAA-3’ |
| At5g25380 | CYCA2;1 | 5’-CGCTTCAGCGGTTTTCTTAG-3’ |
|  |  | 5’-ATCCTCCATTGCAAGTACCG-3’ |
| At5g11300 | CYCA2;2 | 5’-TGTATGTGTTGGCCGTAATG-3’ |
|  |  | 5’-TGGTGTCTCTTGCATGCTTA-3’ |
| At1g15570 | CYCA2;3 | 5’-TTCCTCCATGGAAACTACGTG-3’ |
|  |  | 5’-CGTTTTTGGAGTGGGAGTGTA-3’ |
| At1g80370 | CYCA2;4 | 5’-GACAGCAGTGATGGTACCTCCTTTCTGCC-3’ |
|  |  | 5’-CCTGCGTGTTGCTGAGCTTAAACGAAGAC-3 |
| At5g43080 | CYCA3;1 | 5’-GATACAAAGCGGGTGATTTGA-3’ |
|  |  | 5’-AGAGGCAGCTCTGGTGATACA-3’ |
| At1g47210 | CYCA3;2 | 5’-TTTGCAGCTAGAGCCTCTTTG-3’ |
|  |  | 5’-GATCAGCCGCTTTGTACTTTG-3’ |
| At1g47220 | CYCA3;3 | 5’-GGGTTGCACAAGAAGATTTCA-3’ |
|  |  | 5’-ATGAATCGAGCGAGAAAGACA-3’ |
| At1g47230 | CYCA3;4 | 5’-CGCCATGACCTAAAAATCTCA-3’ |
|  |  | 5’-CCTCGCACAGTTCTGATTCTC-3’ |
| At4g37490 | CYCB1;1 | 5’-CACGTCTACTACCTTTGGTTTCC-3’ |
|  |  | 5’-GATCAATCATCGTCCTCGTACAC-3’ |
| At5g06150 | CYCB1;2 | 5’-TCAGTGCCTTGCTTATTGCTTCC-3’ |
|  |  | 5’-GTCGGGACTGTCAAATACCATTCG-3’ |
| At3g11520 | CYCB1;3 | 5’-GCAAGAGGTGATAAGCGTGAG-3’ |
|  |  | 5’-CTTGCTTCGAGCATCAAGAAC-3’ |
| At2g26760 | CYCB1;4 | 5’-CGTGGAATCGCAGGTGAAATCAAACCG-3’ |
|  |  | 5’-GTCCACTCGCAGCCTTGCTTCGAGCTC-3’ |
| At2g17620 | CYCB2;1 | 5’-AAGTGCTAACGACTTCGGTGA-3’ |
|  |  | 5’-CGATCGGTTCTTCCACTGTTA-3’ |
| At4g35620 | CYCB2;2 | 5’-GGAGAGCATTGGGTGTGATTA-3’ |
|  |  | 5’-CTCGTTTCCACTTGGTTTCAG-3’ |
| At1g20610 | CYCB2;3 | 5’-TAAACCACCTGTGCATCGAC-3’ |
|  |  | 5’-ATCTCCTCCAGCATTGCTTC-3’ |
| At1g76310 | CYCB2;4 | 5’-CGACAACAAGGGGGTTTACGTGGAGG-3’ |
|  |  | 5’-GTCTGGTTTCTTGGTTTCCTCCTTGTGG-3’ |
| At1g70210 | CYCD1;1 | 5’-GTCGCATGGATTCTCAAGGTA-3’ |
|  |  | 5’-TTTCCTCCATCTTTGCAGCTA-3’ |
| At2g22490 | CYCD2;1 | 5’-CCTCCTCAGAAATCTCCCATC-3’ |
|  |  | 5’-AATTCGTTTATTGGGGTGGTC-3’ |
| At4g34160 | CYCD3;1 | 5’-CACTGGGATTTCCTCAACAAA-3’ |
|  |  | 5’-AAAGAGGGTCAAAGGGATCAA-3’ |
| At5g67260 | CYCD3;2 | 5’-AGCACAATCCAAGCAAGAAGA-3’ |
|  |  | 5’-ATCTCATTTGCTGCTCCTGAA-3’ |
| At3g50070 | CYCD3;3 | 5’-CGCTTACATTTTCAAGTGGAAGAAGCAAG-3’ |
|  |  | 5’-CTCAGAAATCTCGAATCAGGAATAATGG-3’ |
| At2g36010 | E2Fa | 5’-CCTCAACCCGGCGATACCTC-3’ |
|  |  | 5’-GGTGGAGTACTCACGTCGGC-3’ |
| At5g22220 | E2Fb | 5’-ACTTTGCACAGGCCGGTACT-3’ |
|  |  | 5’-GACATCGAGACCCTTCCACTGA-3’ |
| At2g28740 | Histone H4 | 5’-TTAGGCAAAGGAGGAGCAAA-3’ |
|  |  | 5’-CTCCTCGCATGCTCAGTGTA-3’ |
| At4g22910 | CCS52A1 | 5’-CACGCTGCAAGAGAACAAGA-3’ |
|  |  | 5’-ACCACTTGAGTCCGCATACC-3’ |
| At5g08550 | ILP1 | 5’-AGCTTGCCAAGAAGGCATTG-3’ |
|  |  | 5’-TCATCAACGACGCAGTCAGA-3’ |
| At1g25580 | SOG1 | 5’-GACAACAGTGATGTTGTTGATGATTGG-3’ |
|  |  | 5’-CCATCACTCTTAACACCTGGTAAATTC-3’ |
| At3g22370 | AOX1a | 5’-CATGGAAGTCGCGAAACCGA-3’ |
|  |  | 5’-CGAAGTGTCGCATCAGCAGGAAGCC-3’ |
